# Prevalence of wasting and associated factors among children aged 2–5 years, southern Ethiopia: a community-based cross-sectional study

**DOI:** 10.1186/s40795-022-00657-x

**Published:** 2022-12-30

**Authors:** Helen Ali Ewune, Reta Kassa Abebe, Daniel Sisay, Getanew Aschalew Tesfa

**Affiliations:** 1 Human nutrition department, School of Public Health, Dilla University, Dilla, Ethiopia; 2 School of Public Health, Dilla University, Dilla, Ethiopia

**Keywords:** Acute malnutrition, Children, Ethiopia, Gedeo, Wasting

## Abstract

**Background:**

Wasting (acute malnutrition) is the most serious form of malnutrition for children in the near term. Malnutrition has a variety of causes, all of which are interconnected and hierarchically related. The purpose of this study was to assess the prevalence of wasting and its associated determinants among children under the age of five in the Wonago district, Gedeo zone, southern Ethiopia.

**Methods:**

Community based cross-sectional study was conducted from October 1 to 30, 2021 using a systematic random sampling technique. Data were entered using Epidata manager and STATA v.20 software was used for analysis. Descriptive statistics were reported to describe the study population. To identify associated factors of wasting, bivariate and multivariate logistic regression analysis were fitted. Variables having *p*-value < 0.05 were declared statistically significant predictors of wasting.

**Results:**

A total of 390 respondents participated with a response rate of 92.6%. The prevalence of wasting among children aged 2–5 years in Wonago district was 36.4% (95% CI: 31.76–41.32). Moderate household food insecurity (AOR = 0.35, 95%CI: 0.14–0.83), history of recurrent illness (AOR = 0.15, 95%CI: 0.26–0.84), and duration of breastfeeding greater than 2 years (AOR = 0.15, 95%CI: 0.26–0.84) were significantly associated with wasting.

**Conclusion:**

Almost one-third of the children were wasted. Household food insecurity, breastfeeding, and recurrent illness were significantly associated with wasting among children aged 24–59 months. It is recommended that interventions be designed with food security, disease prevention, and breastfeeding awareness in mind and put the spotlight on food and nutrition policy to insure children’s nutritional status.

**Supplementary Information:**

The online version contains supplementary material available at 10.1186/s40795-022-00657-x.

## Background

Children’s nutritional status is a significant measure of a household’s living standard and a factor of child survival, as well as a powerful reflection of a country’s growth [1]. Malnutrition is a condition in which an individual’s physical function is damaged to the degree that she or he is unable to sustain appropriate body performance processes such as growth, pregnancy, lactation, physical work, and illness resistance and recovery [2–4].

The World Health Organization (WHO) recommends child malnutrition as one of the health indicators for health equity [5]. Stunting, wasting, and underweight are frequent anthropometric measures used to assess malnutrition in children under the age of five. Underweight (low weight-for-age) represents both low height-for-age and low weight-for-age, and so chronic and acute malnutrition exposures [6]. In the short term, wasting (acute malnutrition) is the most dangerous type of malnutrition for children. Also known as weight for height with a z-score less than minus two standard deviations from the median weight for height in the standard reference population [7, 8].

Under the age of five is a critical developmental period because it represents the transition from childhood to adolescence, spanning the ages of 0–59 months [9].

In developing countries, malnutrition is still a problem for public health. In particular, among young children under the age of five, malnutrition in various forms, such as acute malnutrition or wasting, stunting, and underweight, is still widespread. People all over the world are impacted by it. Currently, it is held responsible for 2.3 million children’s deaths, or more than 41% of all fatalities among children under the age of five in developing countries [10].

Wasting still affects 50.5 million children under the age of five globally, with 17 million seriously wasted. The highest prevalence of undernutrition in the world is estimated to affect 264.2 million people in sub-Saharan Africa, or 24.1% of the population, in 2020 [11]. Ethiopia has the highest rate of malnutrition among children under the age of 5 years among Sub-Saharan and East African countries, with 9 and 8.7% of wasted under-fives. It is also one of the leading causes of disease and death among children under the age of five in the country [12].

The causes of malnutrition are numerous and multifaceted which causes are intertwined with each other and are hierarchically related. Poor food and sickness are the immediate determinants which are caused by a number of underlying factors, including household food security, maternal/child care practices, access to health care, and a healthy environment. The basic socio-economic and political situations have an impact on these underlying factors [2]. Diseases with an environmental component, such as those spread by insect or protozoan vectors, or those induced by a micronutrient-deficient environment, plainly impact nutritional health [7].

Despite the efforts of the nongovernmental and government organizations to improve infant and young child feeding (IYCF), wasting among children 2–5 years of age remains a concern in Ethiopia. Nutritional problems have an impact in Ethiopia by affecting the majority of children with severe wasting that accounts one out of every ten children [13].. According to a study conducted in Gobu Soya woreda east Wollega, 12.5% of preschool children are wasted. Family income, family size, and family education were all found to be strongly linked with acute malnutrition among the socioeconomic variables studied [14]. According to the EDHS 2016 data, 9.7% of children are wasted, 28.7% are underweight, and 44.4% are stunted, with considerable regional variations [6]. According to the Health and Health Related Indicators (HHRI) 2014 report, sever acute malnutrition was the fifth greatest cause of death in Ethiopia, accounting for 6.9% of all deaths among children under the age of five [15].

However, there has been a limited study done in Wonago district that demonstrates the prevalence of wasting among children under the age of two to 5 years. Therefore, this study aimed to assess the prevalence of wasting and associated factors among children aged 2–5 years in Wonago district, Gedeo zone, south Ethiopia.

## Methods

### Study area, period and design

A community-based cross-sectional study was conducted in Wonago town, Gedeo zone, South Nation Nationality and People Region (SNNPR), Ethiopia from October 1 to October 30, 2021. The numbers of population in Wonago town is around 156,481. Wonago is located 102, 13, and 377 kms from the zonal, regional and national capitals Hawassa, Dilla, and Addis Ababa, respectively. is bounded by southwest by Yirgachefe District, northwest by the Oromia region, northeast by Dilla Zuriya District, and southeast by Bule District. The town has three Kebeles in which 1737 children aged 2–5 years is found.

### Study population

#### Source population

All children aged 2-5 years living in Wonago district.

### Study population

Selected children aged 2–5 years paired with their mother/care givers living in Wonago district.

Study Unit.

Selected children aged 2-5 years paired with their mother/care givers living in Wonago district and participated in actual response during data collection period.

Eligibility All 2–5 children paired with their mother/care givers living in Wonago district.

### Sampling technique and procedure

#### Sample size determination

The sample size was calculated using single population proportion formula by considering the following assumptions: *p* = 0.476 proportion of wasting in Bulle Hora town with the level of confidence = 95%, level of significance = 5% and margin of error (d) =5%. *Therefore, the final total sample size including the non-response rate was* *421.*

### Sampling procedure

**Fig. 1:**
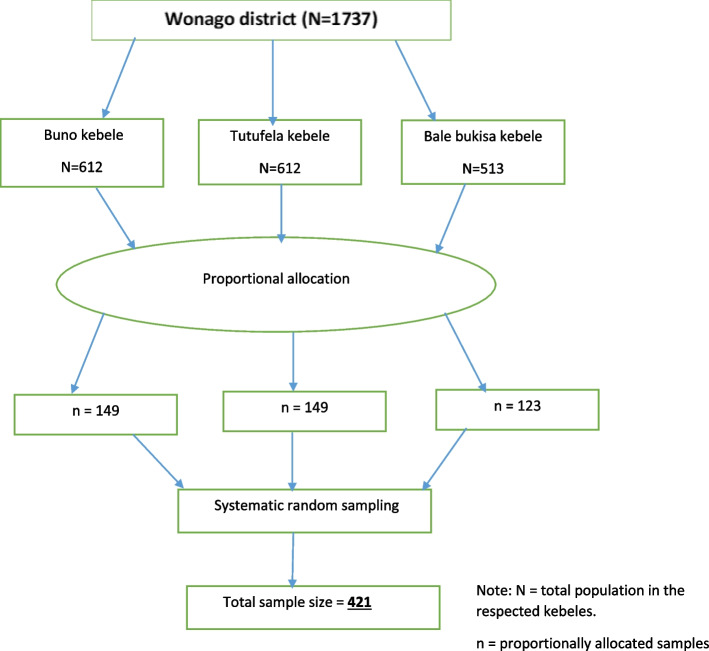
Sampling procedure of the study

A systematic random sampling technique was used. Based on the proportional allocation formula 421 study participants were distributed to the three kebele of wonago town and the kth value was calculated (Kth = 4) (Fig. 1). The first house hold with 2–5 years child in each kebeles was selected randomly from 1 to 5 households by lottery method then the rest was selected every respected 4 household until the total sample size was achieved.

### Operational definitions

- Wasted:- A child weight for height z score < −2sd [16]
- Not wasted: A child weight for height z score= > −2sd [16]
- Caregiver: is a mother, father, any family member or paid person that knows about the child in detail and help the child with feeding, dressing, undressing, and with hygiene.
- Food-insecurity: Exists when all people, at all times, lack secure access to sufficient amounts of safe and nutritious food that meets their dietary needs and food preferences for an active and healthy life [17].
- Mildly food insecure (access) household Worries about not having enough food sometimes or often and/or is unable to eat preferred foods [17].
- Moderately food insecure household Sacrifices quality more frequently, by eating a monotonous diet or undesirable foods sometimes or often [17].
- A highly food insecure household Experience forced cutting back on meal size or number of meals often, and/or experiences any of the three most severe conditions [17]. Data Collection Procedures and data quality control

### Data collection tool and procedure

A semi-structured interview administered questionnaire, which includes all the relevant information to meet the objectives of the study was used to collect the participants’ Socio-economic factors (child age, family size, income, maternal/ paternal education, Household food insecurity), recurrent illness, Child caring practice (feeding, immunization, Dietary diversity), Maternal characteristics: (age of mother, number of children, ANC visit, age at first pregnancy), environmental health conditions, and dietary diversity practice. The tool was adapted from the world health organization instrument for stepwise surveillance of **(**WHO steps) child malnutrition. In accordance with the steps manual, a few additional questions were added to supplement the questionnaire and to reflect the local context of Ethiopia. It was first written in English, translated into Amharic then to Gede’offa (local language), and was translated back into English by professionals who speak both languages for keeping consistency. Lastly, a survey was conducted with the help of health extension professionals checking intra and inter-professional reliability following the selection of eligible households.

### Data quality control

Data was gathered by 15 data collectors who had been trained. Supervisors examined the obtained data on a daily basis for completeness and consistency. Those who can take anthropometric measurements of a child gathered the data. Before measuring, the child’s shoes, braids, and hair clips were removed to reduce measurement mistakes. For height and weight measurements, a stadiometer and a digital weighing machine were utilized. The child was in a standing position throughout the anthropometric measurements. With their feet together and flat on the ground, their heels pressed against the stadiometer’s back plate, their legs straight, buttocks against the backboard, scapula against the backboard, and arms at their sides. Body weight was measured to the nearest 0.1 kg using a stadiometer. The ‘Technical Error of the Measurements’ was calculated by estimating the average difference between the expert’s measurements and those of the trained data collectors, as well as the difference between the data collectors’ first and second measurements (TEM). The relative TEMs for inter and intra examiners relative values for height and weight were greater than 0.95, the suggested cut-off, indicating that the measurements in the study were highly accurate.

Before the actual data collection took place, pretest was done on the sample of 5% of the sample size, which was in adjacent Woreda to ensure the validity and reliability of methodology and survey tools. Based on the findings of the pretest, the tool was modified. After checking for completeness, the collected data was edited to exclude errors, re-organized, coded and entered into epidata version 4.6 for double data entry verification (to identify data consistency), then was exported to STATA version 20 for windows for cleaning and statistical analysis.

### Data processing and analysis

Epidata version 4.6 was used to enter the data. It was examined for completeness, consistency, and coding using STATA version 20 before any statistical analysis. The WHO Antero plus 2021 version 3.2 software was used to convert anthropometric data into z-scores for the indices stunting HAZ (height for age z-score), underweight WAZ (weight for age z-score), and wasting WHZ (weight for height z-score), and then exported to STATA version 20 for further analysis.

STATA was used to describe the study population using descriptive statistics such as frequency distribution, mean, and proportion. The presence of connections between the various independent predictors and the dependent variable was investigated using bivariate and multivariable logistic regressions. In the bivariate analysis, variables having a *p*-value of less than 0.25 [18] were put into multivariable logistic regression. Variables with a p-value of less than 0.05 in the multivariate analysis were declared statistically linked with wasting in children aged 2 to 5. Finally, conclusions were drawn based on the findings.

### Ethical consideration

The study was conducted after ethical clearance was obtained from the DU review board. Oral consent was gained from the study participants before they were enrolled in the study and anybody involved in this study was informed that she or he has full right to leave the study. The information collected from the respondents were used only for the study purpose.

## RESULTs

### Socio-demographic characteristics

A total of 390 respondents participated with a response rate of 92.6% and the mean age of children was 36 months + 0.02. Among the total participants, of the household heads, 348 (89.23%) were male and 42 (10.77%) were female. Regarding marital status, the majority of the mothers or care givers were married 353 (90.51%) and 37 (9.49%) were single. Concerning the educational status of the mother/ care giver, 128 (33%) were illiterate. Regarding, maternal / care giver occupational status, 11(2.82%) were students, 273 (70.00%) were housewife, 49 (12.56%) were government employees, 57 (14.62%) were merchant. 169 (43.33%) had family size of 2–5 and 221 (56.67%) had family size more than 5(Table 1).

**Table 1:** Socio demographic and economic characteristics of respondents

Variables	Categories	Wasting		Frequency	Percentage
		Yes	No		
Sex of house hold head	Male	125	223	348	89.23%
	Female	14	25	42	10.77%
What did you gave?	Single	21	16	37	9.49%
	Married	227	126	353	90.51%
Age of the mother	15–19 years	2	3	5	1.28%
	20–24 years	13	26	39	10.00%
	25–29 years	54	98	152	38.97%
	30–34 years	38	64	102	26.15%
	35–40 years	35	57	92	23.59%
Paternal occupational status	Farmer	47	76	123	33.24%
	Government employee	22	50	72	19.46%
	Merchant	72	76	121	32.70%
	Private worker	5	8	13	3.51%
	Daily laborer	12	26	41	11.09%
Livestock	Present	46	82	128	32.82%
	Absent	96	166	262	67.18%

### Child characteristics

Among the total study participants, 57(14.62%) of them gave pre-lactational food and 333(85.38%) of them didn’t gave pre-lactational food. of those who took pre-lactational food 35(8.97%) of them took butter, 2(0.51%) of them took milk, 27(6.92%) of them took water. Regarding complementary feeding 34(8.72%) of them started complementary feeding in less than 6 months, 304(77.95%) started at 6 month, and 52(13.33%) of them started after 6 months. Of the respondents 149(38.21%) used bottle to feed their child and 109(33.08%) used cup and 112(28.72%) of them used spoon. Regarding the care of the child, 363(93.08%) were cared by their mother, 20(5.13%) by sister, 5(1.28%) by grandmother, and 2(0.51%) were by housemaids. 73(18.72%) of the participants gave home care when the child became sick, 57(14.62%) of them were taken to the traditional healer, and 260(66.67%) took them to a health facility. Concerning how m any times they took their child to the healthy center, 63(16.15%) took 1–5 times, 36(9.23%) 6–10 times, 10(2.56%) > 10, and 281(72.05%) of them has not taken to the health facility (Table 2).

**Table 2:** Child related characteristics

Variables	Categories	Wasting		Frequency	Percentage
		Yes	No		
Have you ever gave prelactational food?	Yes	21	36	57	14.62%
	No	121	212	333	85.38%
What did you gave?	Water	6	21	27	6.92%
	Butter	15	20	35	8.97%
	Milk	1	1	2	0.51%
	No	120	206	326	83.59%
Have you discarded the colostrum?	Yes	11	13	24	6.15%
	No	131	235	366	93.85%
When did you start complementary feeding?	Less than six month	15	19	34	8.72%
	At six month	104	200	304	77.95%
	Greater than six month	23	29	52	13.33%
What do you use to feed the child?	Bottle	55	94	149	38.21%
	Cup	45	84	129	33.08%
	Spoon	42	70	112	28.72%
How long did you breast feed the child?	Less than one year	13	18	31	7.95%
	Up to two year	81	148	229	58.72%
	Greater than two year	17	44	61	15.64%
	Still breast feeding	24	32	56	14.36%
	I don’t know	7	6	13	3.33%
What did you do when the child become sick?	Home care	48	82	73	18.72%
	Traditional healers	94	166	57	14.62%
	Health facilities	142	248	260	66.67%

### Prevalence of wasting and child care practices

**Fig. 2:**
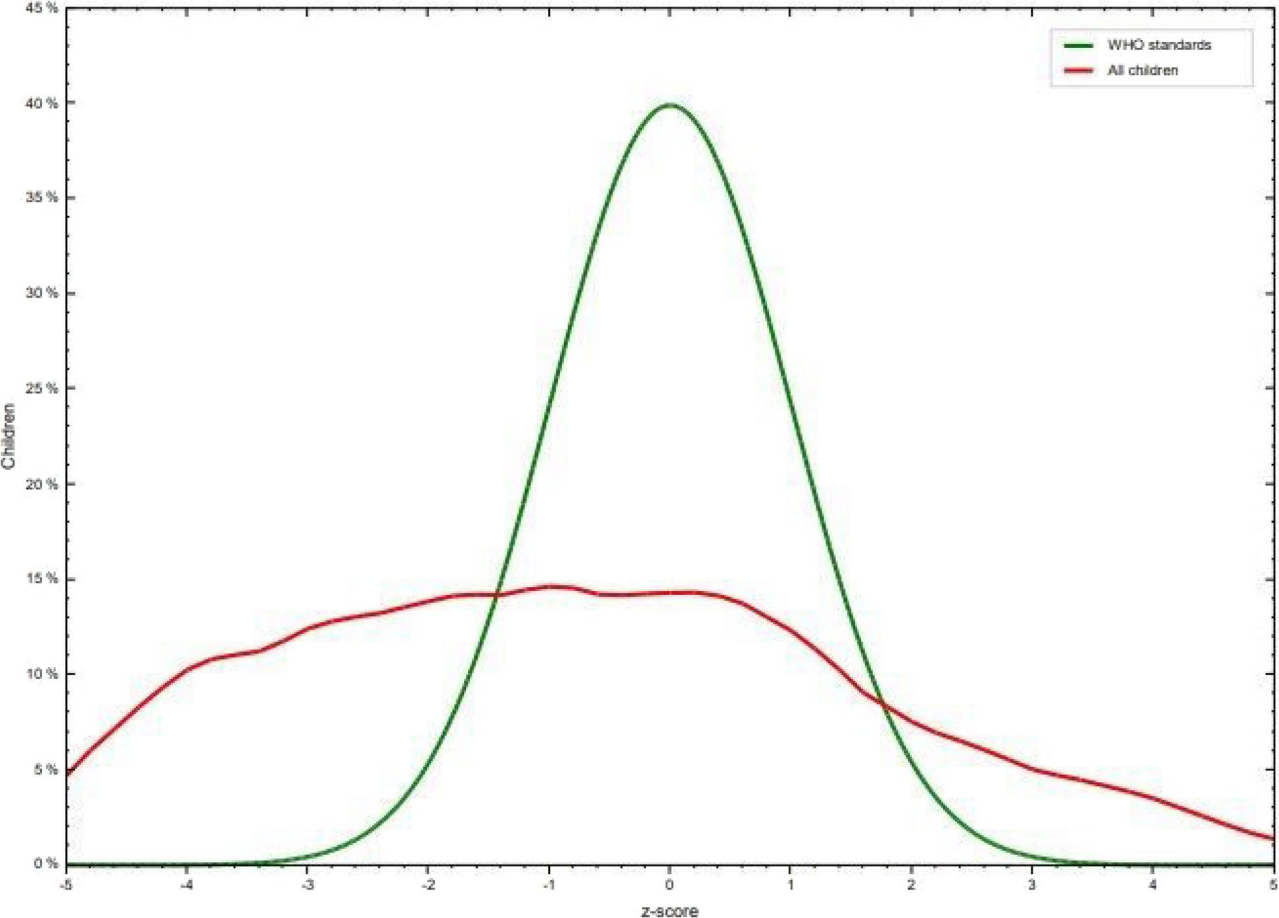
Z score of wasting in comparison with the WHO standard Population

Among the total study participants, 36.41% (95% CI: 31.76–41.32) of them were wasted. 89 (22.8%) children were born at home. In terms of the duration of pregnancy, 13 (3.3%) were below 9 month, 284 (72.8%) were at 9 month, 84 (21.5%) above 9 month, and those who didn’t know were 9 (2.31%). Regarding vaccination status, 11 (2.8%) vaccinated at birth, 17 (4.4%) vaccinated at the 6th week, 21 (5.4%) vaccinated at the 10th week, 29 (7.4%) vaccinated at the 14th week, 24 (6.2%) vaccinated at the 9th month. 139 (35.6%) had recurrent illness and 143 (36.7%) had diarrhea in the past. 34 (8.7%) had measles for the past year (Table 3), (Fig. 2).

**Table 3:** Child care practice

Variables	Categories	Wasting		Frequency	Percentage
		Yes	No		
ANC follow up	Yes	100	204	304	77.95%
	No	42	44	86	22.05%
Family planning	Yes	109	219	328	84.1%
	No	33	29	62	15.90%
Type of family planning	Tablet	14	33	47	12.05%
	Depo	62	107	169	43.33%
	Norplant	34	82	116	29.74%
	Condom	1	1	2	0.51%
	I dint know	31	25	56	14.36%
When do you wash your hand	After toilet	70	111	181	46.41%
	Before preparing food	56	96	152	38.97%
	Before serving food	13	33	46	11.79%
	After washing or taking care of baby	3	8	11	2.82%
How do you wash your hand	Only with water	63	92	155	39.74%
	Sometimes with soap	79	156	235	60.25%

### Maternal characteristics

Mothers who had ANC follow up was 304 (78%) and the number of mothers who utilised family planning was 328 (84.1%), whereas 62 (15.9%) did not utilize family planning. 181 (46.41%) mothers wash their hands after using the restroom, 152 (39.0%) wash their hands before preparing food, 46 (11.79%) wash their hands before serving, and 11 (2.8%) people wash their hands after washing or caring for a baby (Table 4).

**Table 4:** Maternal characteristics

Variables	Categories	Wasting		Frequency	Percentage
		Yes	No		
Place of delivery	Home	31	58	89	22.8
	Health facility	111	190	301	77.17
Duration of pregnancy	Below 9 months	6	7	13	3.3
	At 9 months	102	182	284	72.8
	Above 9 months	29	55	84	21.5
	Don’t know	5	4	9	2.3
Type of delivery	Single	136	246	382	98
	Multiple	6	2	8	2
Vaccination status	At birth	5	6	11	2.8
	at 6th week	6	11	17	4.4
	At 10th week	9	12	21	5.4
	At 14th week	102	186	29	7.4
Recurrent illness	Yes	56	83	139	35.6
	No	86	165	251	64.4
Diarrheal illness in past two weeks	Yes	54	94	148	36.7
	No	88	154	242	62
Respiratory illness in past two weeks	Yes	27	39	66	16.9
	No	112	205	317	81.3
	Don’t know	3	4	7	1.8

### Environmental conditions

River water was used as a source of water by 29 (7.44%) of the respondents. Traditional private pit latrines with wooden slab was the commonest type 181(46.41%). Regarding solid waste disposal, about 146(37.44%) of the respondents dispose garbage on open field, 89(22.82%) dispose the waste by burning, whereas 113(28.97%) dispose in a pit and about 42(10.77%) used the wastes as a fertilizer. About 264(67.69%) of HHs have no separate kitchen for cooking. Out of the total households 368 used woods, 11 used electric, 8 used kerosene, and 3 used biogas as their cooking fuel (Table 5).

**Table 5:** Environmental conditions

Variables	Categories	Wasting		Frequency	Percentage
		Yes	No		
Source of drinking water	Public tap water	94	70	164	42.05
	Private tap	109	47	156	40
	Pond	27	14	41	10.51
	River water	18	18	29	7.44
Water treatment practice	Yes	72	143	215	55.13
	No	70	105	175	44.87
Do you have latrine	Yes	131	228	359	92.05
	NO	11	20	31	7.94
Solid waste disposal	Open area	54	92	146	37.44
	In a hole	33	80	113	28.97
	Use as a fertilizer	18	24	42	10.77
	Burning	37	52	89	22.82
Type of house floor	Soil	98	171	269	68.97
	Cement	39	69	108	27.69
	Wood	5	7	12	3.08
	Ceramic	0	1	1	0.26
Have window for their house	Yes	130	225	355	91.03
	No	12	23	35	8.97

### Factors associated with wasting

First, bi-variate analysis was done and variables with *p*-value of less than 0.25 were included for multivariate analysis. In the multivariate analysis variable having *p* value of < 0.05 were declared statistically significant predictors of wasting.

Household food insecurity, recurrent illness, and duration of breast feeding were significantly associated with wasting among children aged 2–5 years. Children in a households with moderate food insecurity were 65% (AOR = 0.35, 95%CI: 0.14–0.83 *P* value of 0.03) times lower risk of having wasting compared to Children in a households with highly food insecure households. Those children who had no history of recurrent illness were 85% (AOR = 0.15, 95%CI: 0.26–0.84, P value of 0.02) less likely to develop wasting when they are compared to children who had history of recurrent illness. Duration of breast feeding for more than 2 years was found to be 55% (AOR = 0.15, 95%CI: 0.26–0.84 *p* value of 0.017) protective from wasted when compared with breastfeeding less than 2 years (Table 6).

**Table 6:** Bi-variate and multivariate logistic regression analysis of factors associated with wasting

Variables	Categories	Wasting		COR(95% CI)	AOR(95% CI)	***P*** value
		Yes	No			
Household food insecurity	Mild food insecure	38(26.8)	70(28.2)	0.36(0.16–0.79)	0.49(0.20–1.15)	
	Moderate food insecure	37(26.9)	88(35.5)	0.28(0.12–0.61)	0.35(0.15–0.82)^a^	0.03
	Food secure	46(32.4)	76(30.7)	0.40(0.18–0.87)	0.66(0.27–1.52)	
	Highly insecure	21(14.8)	14(5.7)	1	1	
Time of complementary feeding initiated	< 6 months	15(10.6)	19(7.7)	1	1	
	at 6 months	104(73.3)	200(80.7)	0.65(0.32–1.34)	0.90(0.40–2.04)	
	> 6 months	23(16.2)	29(11.7)	1(0.42–2.39)	1.35(0.68–2.66)	
Duration of breast milk fed	< 2 year	101(71.1)	172(69.4)	1	1	
	> 2 years	41(28.9)	76(30.7)	0.91(0.09–1.12)	0.45(0.21–0.96)^a^	0.017
General health checkup	Yes	26(18.3)	55(22.2)	1	1	
	No	116(81.7)	193(77.8)	1.27(0.75–2.13)	1.26(0.68–2.34)	
ANC follow up	Yes	100(70.4)	204(82.3)			
	No	42(29.6)	44(17.7)	1.94(1.19–3.16)	1.18(0.63–2.21)	
Recurrent illness	No	136(95.8)	246(99.2)	0.18(0.03–0.92)	0.15(0.26–0.84)^a^	0.02
	Yes	6(4.2)	2(0.8)	1	1	
Family planning	Yes	109(76.7)	219(88.3)	1	1	
	No	33(23.2)	29(11.7)	2.28(1.32–3.95)	1.72(0.88–3.35)	
Water treatment Practice	Yes	72(50.7)	143(57.7)	1	1	
	No	70(49.3)	185(42.3)	1.32(0.87–2)	1.26(0.77–2.07)	
Waste disposal	Open area	54(38.0)	92(37.1)	1	1	
	Inside hole	33(23.2)	80(32.3)	0.70(0.41–1.18)	0.73(0.41–1.30)	
	As fertilizer	18(12.7)	24(9.7)	1.27(0.63–2.56)	1.19(0.54–2.63)	
	Burning	37(26.1)	52(21.0)	1.21(0.70–2.07)	1.14(0.62–2.09)	

*AOR* Adjusted odds ratio, *COR* Crude odds ratio, *CI* Confidence interval, ^a^significant *p* value < 0.05, 1 = reference

## Discussion

This study was primarily intended to estimate the prevalence of wasting (acute malnutrition) and identify the potential predictors of wasting among children aged 2 to 5 years. A total of 390 children were recruited from three kebeles. Wasting is still prevalent in many impoverished nations, including Ethiopia. According to this study, acute malnutrition is an issue in the Wonago district, and it affects children aged 2 to 5 years.

Our study revealed that the prevalence of wasting was 36.4% (95% CI: 31.23–40.76), which is higher when compared to a study conducted in Mongolia, nearly one out of every five children (22%) suffers from wasting [1]. This could be related to differences in study area, socioeconomic features, health service delivery, and study location. The percentage of wasted children in the current study was also higher than the national level reported by the Ethiopian Demographic and Health Survey (EDHS) 2011, EDHS 2016, and EDHS 2019 [19–21]. This holds with regional levels too, which could be attributable to sample size variations.

In comparison to a study performed in three districts of Nepal’s hilly region (Lamgung, Tanahu, and Gorkha), our study found an increase of 11% among children under the age of five [22]. This could be attributable to the research area and variation of residents’ nutritional status. South Asia has the greatest rate of malnutrition of any region; according to the 2018 global nutrition report, 23% of children are wasted, which is lower than our findings [9].

It is observed to be greater when compared to similar studies conducted in different parts of Ethiopia. In a community-based cross-sectional study conducted in rural kebeles in Haramaya district, 14.1% were wasted. The high prevalence of wasting status could be ascribed to unregulated drinking water sources, which could contribute to various infections, as well as the data collection period, which runs in October, when most homes are short on food.

Despite the positive results and continued efforts to prevent and reduce under-five malnutrition, Ethiopia continues to struggle with undernutrition as a major health issue. In the multilevel analysis, only household food insecurity, recurrent illness, and breastfeeding status were identified as a significant predictors of wasting among children aged 2 to 5 years at *p*-value of < 0.05. The findings of this study reveal that children from families with a moderate level of food insecurity are less likely to be wasted than children from families with a high level of food insecurity. When compared to families with highly food insecure, families with moderate food insecurity had more opportunities to access multiple food groups, which might be the possible reason [23]. Indeed, seasonal variations in food supply, acute food shortages, changes in socioeconomic policy, and diseases developing at higher levels than expected are all linked to wasting [24–26].

Children with recurrent illnesses had a higher risk of wasting in the current study than children who did not have recurrent illness. Supported by the previous study done in Burkina Faso [27]. This could be due to the fact that infection leads the body to lose more energy, predisposing children to wasting. Recurrent acute illnesses are a significant contributor to linear growth retardation [28]. The study also revealed that children who breastfeed for more than 2 years are less likely to develop wasting than children who breastfeed for less than 2 years. This is in line with a study conducted in Kenya, [29] which found that children who had discontinued breastfeeding were likely to be wasted.

### Strengths and limitations

Anthropometric data collection training was taken to reduce technical measurement errors. Additionally, the study was done at the community level which increases the generalizability of the study. Besides it strength, this study has some limitations. Laboratory investigation and related data were not collected in this study. Recall biases could exist among respondents answering questions relating to events happening in the past.

## Conclusion and recommendations

The proportion of wasting was higher as compared to the national figure. Almost one-third of the children were wasted. Moderate household food insecurity, breastfeeding greater than 2 years, and recurrent illness were significant identifying variables of wasting among children 24–59-months age group. To increase household food security and provide a long-term solution for this problem, the local administration must collaborate with other governmental and non-governmental organizations. Likewise, it is recommended that interventions be designed with food security, disease prevention, and breastfeeding awareness in mind and putting the spotlight on food and nutrition policy to insure children’s nutritional status.

## Supplementary Information

**Additional file 1.** 

## Data Availability

The datasets supporting the conclusions of this article are included within the article. All authors agreed to share the raw data hence, the datasets generated and/or analyzed during the current study are available as a supplementary file.

## Abbreviations

ANC: Ante Natal Care
AIDS: Acquired Immune Deficiency Syndrome
COHA: Cost Hunger in Africa
DU: Dilla University
EDHS: Ethiopian Demographic and Health Survey
EPI: Expanded program on Immunization
HIV: Human Immune Virus
IYCF: Infant and Young Child Feeding
MAUC: Mid upper arm circumference
SAM: Severe Acute Malnutrition
SD: Standard Deviations
SNNP: South Nation Nationalities and peoples
UNICEF: United Nation for children’s fund
WHO: World Health Organization
